# The Whole Macular Choroidal Thickness in Subjects with Primary Open Angle Glaucoma

**DOI:** 10.1371/journal.pone.0110265

**Published:** 2014-10-28

**Authors:** Shunsuke Nakakura, Minami Yamamoto, Etsuko Terao, Toshihiko Nagasawa, Hitoshi Tabuchi, Yoshiaki Kiuchi

**Affiliations:** 1 Department of Ophthalmology, Saneikai Tsukazaki Hospital, Himeji, Japan; 2 Department of Ophthalmology and Visual Sciences, Graduate School of Biomedical Sciences, Hiroshima University, Hiroshima, Japan; Massachusetts Eye & Ear Infirmary, Harvard Medical School, United States of America

## Abstract

**Purpose:**

We investigated the whole macular choroidal thickness in subjects with glaucoma in order to evaluate the effects of glaucoma and glaucoma visual field damage on the choroidal thickness.

**Subjects and Methods:**

We examined 40 primary open angle glaucoma patients with only superior visual field defects and 48 normal controls. The macular choroidal thickness was measured using swept-source optical coherence tomography according to the three-dimensional raster scan protocol (6×6 mm). We used the choroidal thickness within a 1.0-mm circle measured on ETDRS grids as the central sector and then used a 6×6 rectangular grid to divide the area into six sectors.

**Results:**

No significant differences were found in the choroidal thickness values between the glaucoma and normal subjects in any of the sectors after adjusting for the age and axial length (all P>0.4, ANCOVA). According to a stepwise analysis of the glaucoma subjects performed using the parameters of age, axial length, central corneal thickness and mean deviation (MD value) obtained by static perimetry, age was the most predictive and significant factor in all sectors (coefficient  = −3.091 to −4.091 and F value  = 15.629 to 22.245), followed by axial length (coefficient  = −10.428 to −23.458 and F value  = 2.454 to 6.369). The central corneal thickness and MD values were not significant predictive factors in any of the sectors. No significant predictive factors were found for the differences in the choroidal thickness values observed between the superior and inferior field sectors.

**Conclusions:**

Neither the glaucoma-related visual field damage nor glaucoma itself have any apparent associations with the whole macular choroidal thickness.

**Trial Registration:**

Japan Clinical Trials Register (http://www.umin.ac.jp/ctr/ number, UMIN 000012527).

## Introduction

Whether the choroidal thickness is truly associated with the etiology or progression of glaucoma remains unknown. The uvea (choroid, iris and ciliary body) contains abundant large and small vessels, and utilizes more than 80% of the ocular blood flow [1]. The factors that influence the ocular blood flow, such as a low systolic perfusion pressure, low systolic blood pressure and history of cardiovascular disease, have been reported to be predictors of long-term glaucoma progression [2]. Recently, the development of enhanced-depth imaging optical coherence tomography (OCT) based on spectral-domain OCT has enabled clinicians to measure the choroidal thickness noninvasively. Previous studies have reported that a thinner choroidal thickness value at the fovea is associated with an older age [3]–[9], longer axial length [3]–[6], [8], [9] and thicker central corneal thickness (CCT) [6], [9].

With respect to the association between glaucoma and the choroidal thickness, previous reports have shown no significant differences between glaucoma patients and normal subjects [5], [7], [9] or among individuals suspected of having glaucoma [6] based on the choroidal thickness value. However, Hirooka et al. reported that, in the setting of normal tension glaucoma (NTG), which was diagnosed when there was an untreated peak IOP≤21 mmHg, including the 24-h fluctuations, there was choroidal thinning 3 mm nasal from the fovea compared with that observed in normal subjects [10]. Meanwhile, Usui et al. reported that, in highly myopic eyes (with a spherical equivalent refractive error between -6 and -12 diopters, and an axial length greater than 26.5 mm), NTG patients exhibit thinner choroidal values at the fovea and around the optic disc than subjects without glaucoma [11]. However, even if the choroidal thinning or thickening is associated with glaucoma, whether such changes in the choroidal thickness values are primary or occur secondarily due to the progression of glaucoma remains unknown.

Furthermore, most of the previous reports have focused on the subfoveal choroidal thickness and/or choroidal thickness values at +/− 3 mm nasal or temporal to the fovea. Glaucoma is generally characterized by optic disc changes with corresponding nerve fiver defects and visual field defects. The initial changes most frequently occur in the inferior optic disc rim, corresponding to a superior visual field defect, and it does not exceed the central line, since the normal retinal nerve fiber layers run symmetrically in the superior and inferior fields. However, the fovea is free of a nerve fiber layer, as the inner retina and ganglion cells are pushed away to the foveal slope. Therefore, the use of measurement points at the fovea or +/− 3 mm nasal or temporal to the fovea may not be appropriate for evaluating the relationship between glaucoma and the choroidal thickness.

Swept-source optical coherence tomography (SS-OCT) applies a swept wavelength laser as a light source [12], [13], with a longer center wavelength that penetrates deeply into tissues. The longer wavelength (1,050 mm) of SS-OCT is attenuated by water absorption; however, SS-OCT can achieve much less roll-off, thus leading to retained sensitivity at increasing depths, and can operate at a higher speed than spectral domain OCT to obtain the choroidal thickness maps using the 3D raster scan protocol [14].

The aim of this study was to investigate the whole macular choroidal thickness (6×6 mm) in patients with superior glaucoma visual hemi-field defects in order to: 1) compare these values with those observed in normal subjects after adjusting for the previously identified factors associated with the choroidal thickness (age and axial length) and 2) to investigate whether the degree of glaucoma visual field damage is associated with the choroidal thickness and/or differences in the choroidal thickness values between the superior and inferior sectors.

## Subjects and Methods

We studied 88 subjects (40 glaucoma patients and 48 normal subjects) treated at Saneikai Tsukazaki Hospital from October 2012 to December 2013. This study received approval from the Institutional Review Board of Saneikai Tsukazaki Hospital and was performed according to the tenets of the Declaration of Helsinki. Written informed consent was obtained from each participant prior to enrollment in this study. All subjects received a full ophthalmic examination (conducted by S.N. or T.N) and had a best-correlated visual acuity of more than 0.6, refractive errors (spherical equivalent) within ±6 diopters (D) and cylinder correction within ±3D. The refraction was measured using an autorefractor (KR-8800, Topcon Corporation, Japan). The axial length (AL) was measured using an IOLMaster, ver. 5.02 (Carl Zeiss Meditec, Jena, Germany), and the mean of five measurements was used in the subsequent analyses. The CCT values in the glaucoma subjects were measured with a specular microscope (SP-3000, Topcon Corporation, Japan). The intraocular pressure was measured using a Goldmann applanation tonometer with the subject in the sitting position.

All glaucoma patients had attended our clinic for at least one year and their disease was well controlled with topical anti-glaucoma eye drops. Each patient received a reliable visual field analysis (Humphry Visual Field Analyzer (HFA), Carl Zeiss Inc., Dublin, CA) according to the thresholding algorithm (SITA-standard) 30-2, and underwent HFA at four- to six-month intervals. The visual field test results used in the study were obtained within ± three months of the time when the choroidal thickness was measured, and which had fixation losses less than 20%, and the false-positive and false-negative errors were less than 15%. A glaucomatous visual field defect was defined as 1) a glaucoma hemifield test graded “outside the normal limits” and 2) a cluster of three contiguous points at the 5% level for the pattern deviation plot, with at least one point being P<1%. The mean deviation value (MD value) was used for the analysis. All patients had primary open angle glaucoma, as defined by 1) the results of a gonioscopic examination that revealed an open angle and 2) the presence of visual field defects in least one of the eyes whose locations corresponded to glaucomatous disc excavation, namely, the presence of a focal or diffuse defect of the optic disc rim with or without retinal nerve fiver layer (RNFL) defects.

**Figure 1:**
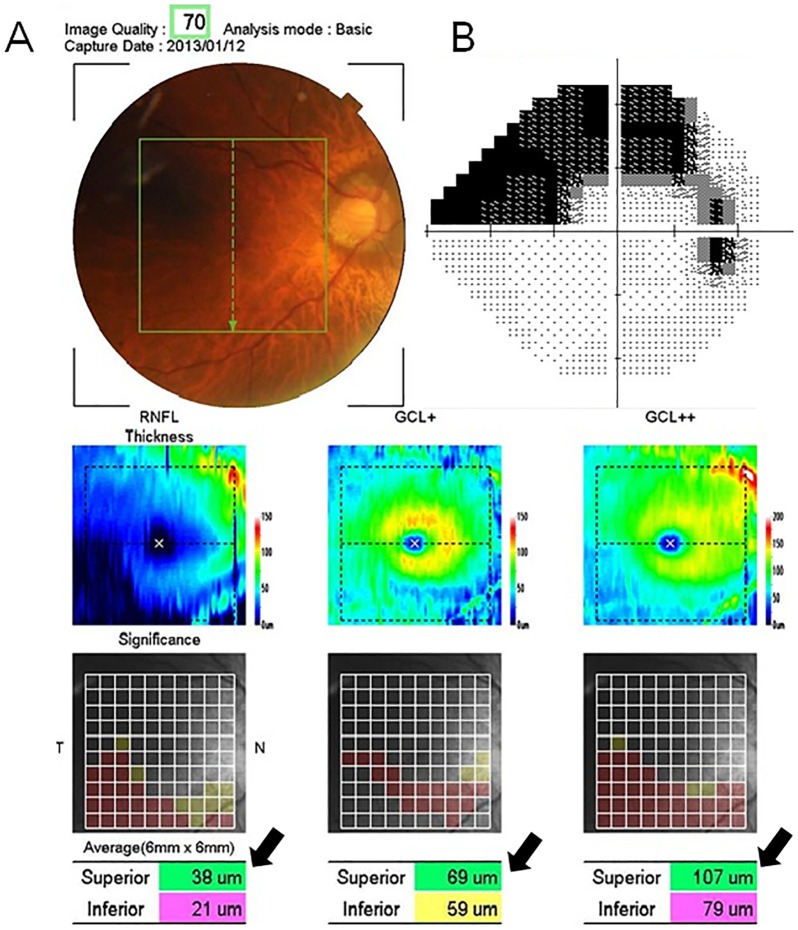
The 3D-OCT map and results of the HFA test in a 71-year-old male subject with glaucoma. (A) The 3D-OCT map provides a 10×10 grid map of the macular RNFL (GCL+), ganglion cell layer (GCL)/inner plexiform layer (IPL) thickness (GCL++) and macular RNFL+GCL+IPL. Upper panel: a pseudo-colored map of the measured thickness. Center panel: Each grid in the 10×10 grid was color-coded, with no color (within the normal limit), yellow (outside of the 95% normal limit) or red (outside of the 99% normal limit) used to indicate different values. Lower panel: the thicknesses of the total, superior and inferior hemiretinal sectors. The black arrows show the superior hemiretinal thickness, which is displayed as color-coded with green (within the normal limit), yellow (outside of the 95% normal limit) or red (outside of the 99% normal limit) based on the software program's normal built-in dataset. (B) The results of the HFA test. The MD was −9.38 dB (P<0.5%) and the PSD was 14.54 dB (P<0.5%). Superior glaucoma visual field damage corresponds to inferior optic disc rim thinning and the 3D-OCT data. The inferior visual field was intact.

In the present study, we recruited glaucoma patients who exhibited only focal or diffuse inferior optic rim thinning with corresponding superior visual field loss. Patients with a history of any previous glaucoma surgery or systemic diseases, such as diabetes mellitus or uncontrolled hypertension, the use of an oral carbonic anhydrase inhibitor (acetazolamide) and/or an unknown previous refraction before cataract surgery were excluded. Patients who displayed any optic disc changes suspected to be due to glaucoma at the superior optic disc rim confirmed based on the presence of ganglion cell-loss on three-dimensional OCT were also excluded (3D-OCT; Topcon, Inc., Tokyo, Japan). The 3D-OCT provides a 10×10 grid map of the macula RNFL and ganglion cell layer (GCL)/inner plexiform layer (IPL) thickness, and uses a software program that displays a deviation map indicating if there is a significant reduction of the total macular retinal thickness, superior hemiretinal thickness or inferior hemiretinal thickness compared to that observed in a built-in normal database in three areas (the mean macular RNFL, mean GCL/IPL and mean macular RNFL+ mean GCL/IPL), with a probability of less than 1% [15]. All glaucoma patients exhibited only inferior hemiretinal thickness abnormalities (displayed in yellow or red), with a normal superior hemiretinal thickness (displayed in green). Figure 1 shows the macular retinal thickness values in the glaucoma patients measured on 3D-OCT and the glaucoma visual field defects measured on HFA (The 3D-OCT data are provided in Table S1).

The inclusion criteria for normal subjects were 1) IOP ≤21 mm Hg 2) normal ophthalmoscopic appearance of the optic nerve (cup-to-disc ratio <0.5 in both eyes, cup-to disc ratio asymmetry <0.2, the absence of hemorrhage, or the presence of localized or diffuse rim thinning).^5^ The exclusion criteria for the normal controls were as follows: 1) a history of intraocular surgery, 2) a history or evidence of chorioretinal or vitreoretinal disease and 3) systemic disease, such as diabetes mellitus or uncontrolled hypertension. Normal subjects did not undergo HFA testing.

### Swept-source Optical Coherence Tomography (SS-OCT)

The macula area (6×6mm) of the eyes was examined with the SS-OCT instrument (DR1 OCT-1; Topcon, Tokyo, Japan). The SS-OCT has an acquisition rate of 100,000 A-scans per second when operated with the 1-µm wavelength tunable laser centered at 1,050 nm, with an approximate 100-nm tuning range and a tissue imaging depth of 2.6 mm [14]. Following pupil dilation, the SS-OCT examinations were performed by experienced certified orthoptists (M.Y and E.T) from 1:00 PM to 5:00 PM to reduce the effects of diurnal fluctuations [16], [17]. The 3D volumetric raster scan protocol was used, which covered an area measuring 6×6 mm centered on the fovea, with 512 A-scans × 256 B-scans. All images had image quality scores ≥45 (of 160), according to the manufacturer's recommendations.

Using a series of 64 B-scan images, each of which was created by averaging four consecutive B-scans, a choroidal thickness map was created via semiautomatic segmentation. The choroidal thickness was measured as the distance from the outer border of the retinal pigment epithelium to the inner surface of the chorioscleral interface. However, semiautomatic segmentation does not always provide the correct chorioscleral interface; therefore, manual segmentation using the built-in software program was performed on all 64 B-scan images in each patient by experienced certified orthoptists (M.Y and E.T). The interobserver reproducibility of the choroidal thickness values obtained using this manual segmentation method has previously been reported by our group, and the ICC for the choroidal thickness values between the two observers was very good (from 0.990 to 0.999) [14].

**Figure 2:**
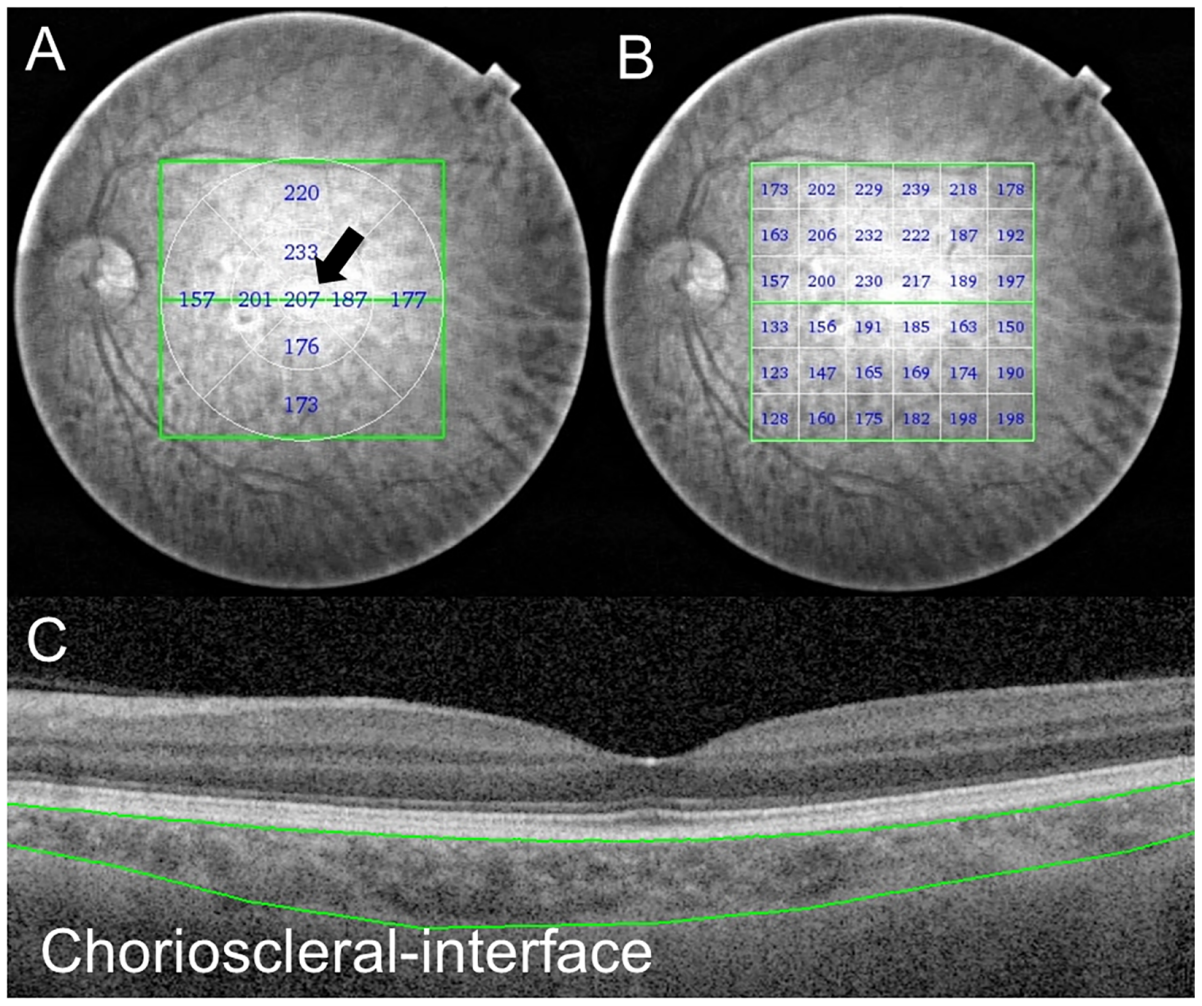
The choroidal thickness map of a 57-year-old normal male obtained using SS-OCT. The 3D raster scan protocol with 512A-scans ×256 B-scans was used to obtain the 3D imaging data for a 6×6-mm area. (A) The black arrow shows the central sector choroidal thickness as measured on the ETDRS grid within a 1×1-mm circular area. (B) The choroidal thickness values presented by the 6×6 rectangular grid map. Each value shows the mean choroidal thickness within a 1×1-mm square area. (C) Manual segmentation of the chorioscleral interface on the B-scan image. All 64 B-scan images were obtained for each subject.

**Figure 3:**
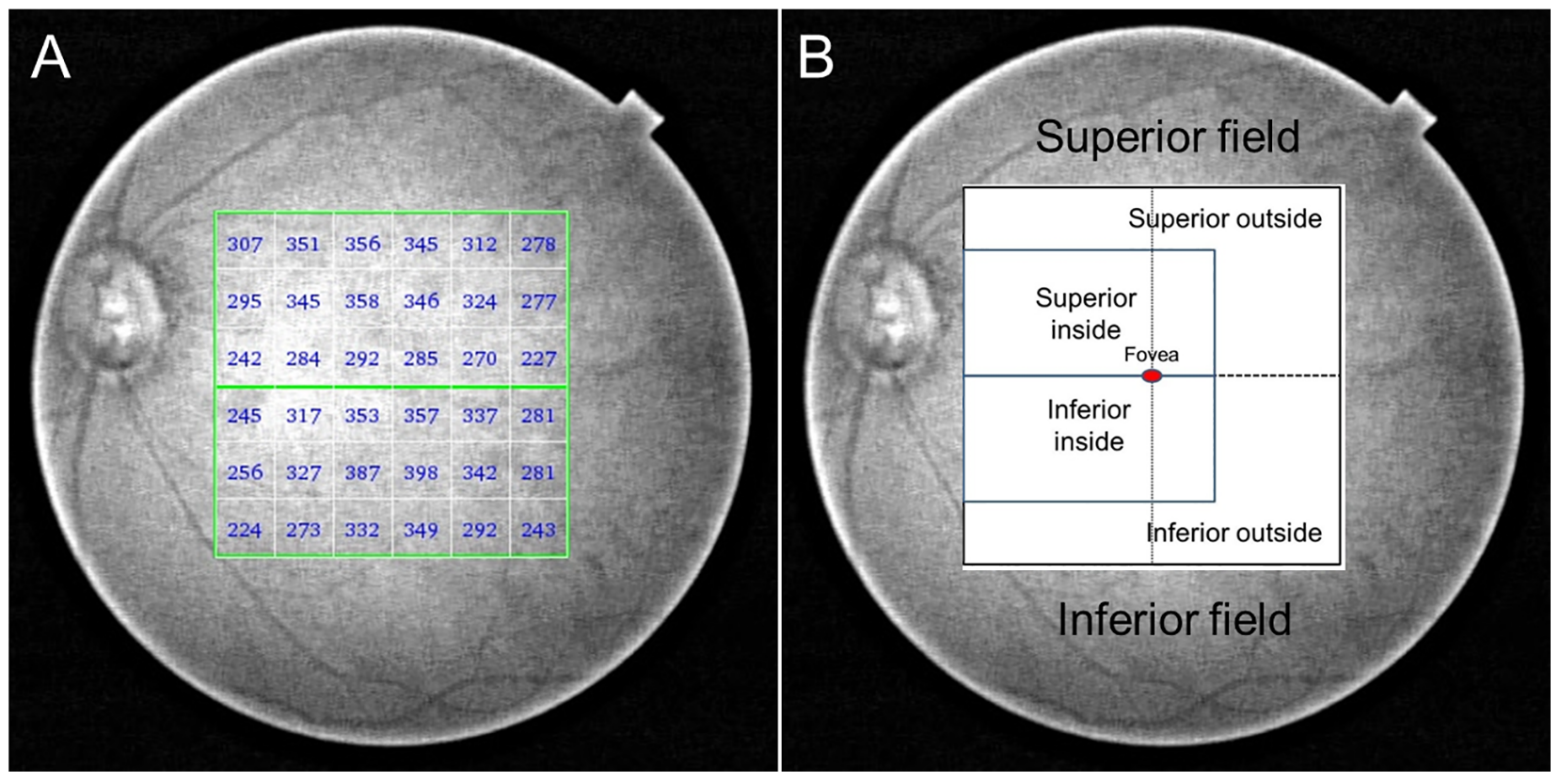
The choroidal thickness map of a 74-year-old female subject with glaucoma obtained using SS-OCT. (A) The choroidal thickness values presented on the 6×6 rectangular grid map. (B) The six sectors used in the present study. Each area was symmetrically divided between the superior and inferior sectors by a horizontal line.

The central sector, the center of the 1.0-mm circle on the ETDRS grid (Figure 2) and the 6×6 rectangular grid (Figure 2) (1×1 mm for each section), was used for the subsequent sector analysis. The 6×6 rectangular grid (36 sections) was divided horizontally into the superior field (18 sections) and inferior field (18 sections). The superior field was divided superiorly inside (including the fovea, eight sections) and outside (the remaining 10 sections), and a similar partition was attempted on the inferior field (Figure 3). This deviation was developed for the present study based on the RNFL lanes.

### Statistical Analysis

We used the JMP, version 10.0.0 software package (SAS Institute Inc., Cary, NC, USA) for the statistical analyses, and the data were expressed as the means ± standard deviation (SD). Values of p<0.05 were considered to be statistically significant. For comparisons of the demographic data between the glaucoma and normal subjects, we used Student's *t*-test for the age, IOP and axial length and the chi-square test for sex and the target eye.

We compared the macular choroidal thickness values between the glaucoma patients and normal subjects using an analysis of covariance, with covariance considered for age and the axial length. We used a stepwise regression analysis to evaluate the significant factors affecting the choroidal thickness and the differences in the choroidal thickness values between the sectors in the glaucoma patients based on the following representative factors: age, axial length, CCT and MD. The independent variable criterion of P = 0.2 was set for the analysis.

The sample size required to detect a 20% (approximately 40 µm) difference between the glaucoma and normal subjects with a significance level of 5% and a power of 80%, based on the standard deviation of 54.8 µm in the inferior field choroidal thickness average in the glaucoma subjects, was estimated to be 30 patients per group.

## Results

The patient demographics of the two groups and the MD values in the glaucoma patients are shown in Table 1. The glaucoma group consisted of more female patients than the normal group (P<0.01). No significant differences were found between the two groups in terms of the age, the target eye, IOP or axial length.

**Table 1:** The patient demographics.

	Glaucoma N = 40		Normal N = 48		
	mean ±SD	range	mean ±SD	range	P value
Age (years)	65.2±11.8	40–85	66.4±11.8	31–87	0.631*
Sex/female (%)	31(78)		24(50)		0.007†
Right eye (%)	19(47)		27(56)		0.413†
IOP (mmHg)	15.8±2.6	12–21	15.6±2.8	9–21	0.832*
Axial length (mm)	23.7±1.2	20.6–26.0	23.9±1.1	21.8–27.6	0.514*
CCT (µm)	502.9±28.9	445–558	-	-	-
Mean deviation (dB)	−5.1±4.7	−19.64–0.24	-	-	-

*, Student's *t*-test

† , chi-square test.

Table 2 shows the results of a comparison of the choroidal thickness values following adjustment for age and the axial length in all sectors, and the p values of the ANCOVA analysis. No significant differences were found between the glaucoma group and the normal group in any sector (P>0.4).

**Table 2:** Comparisons among the sectors between the glaucoma and normal groups.

	Glaucoma	95% CI	Normal	95% CI	P value
The central sector	219.1±11.2	196.7–241.6	216.6±10.2	196.1–237.1	0.869
a: superior field ave	208.6±8.9	190.8–226.5	200.9±8.1	184.6–217.2	0.531
b: inferior field ave	193.2±9.1	174.9–211.5	185.4±8.3	168.8–202.1	0.536
c: superior inside ave	198.8±9.9	179.1–218.5	193.8±9.0	175.8–211.8	0.712
d: superior outside ave	216.7±8.5	199.7–233.6	206.6±7.7	191.1–222.1	0.390
e: inferior inside ave	188.0±10.1	167.8–208.1	183.0±9.2	164.7–201.4	0.720
f: inferior outside ave	197.3±8.7	179.8–214.7	187.4±8.0	171.4–203.3	0.408

Ave, average.

The data are presented as the age- and axial length-adjusted mean ± standard error of the mean (SEM) and 95% CI (confidence interval) (µm).

All P values were obtained with an ANCOVA using the age and axial length as covariates.

The raw data for the choroidal thickness values in the glaucoma group are shown in Table 3, and Table 4 shows a comparison between the superior field sectors and inferior field sectors (symmetrical comparison). The choroidal thickness values were significantly thinner in the inferior sectors in all symmetrical comparisons (P<0.05). We also compared the inside and outside choroidal thickness values, and found that the outside choroidal thickness values were significantly thicker in both the superior and inferior fields (all P<0.05). These data differ from the values in Table 2 because they were obtained without adjustment for age or the axial length.

**Table 3:** The choroidal thickness values in the glaucoma group.

	mean ±SD	range
The central sector	222.6±71.7	77.0–397.0
a: superior field ave	210.4±57.1	103.7–346.8
b: inferior field ave	195.8±54.8	88.3–318.6
c: superior inside ave	202.3±64.1	90.0–359.6
d: superior outside ave	219.8±53.1	119.1–332.6
e: inferior inside ave	190.9±62.6	77.6–355.0
f: inferior outside ave	199.7±50.8	96.9–295.4
difference 1 (a-b)	14.5±29.6	−55.5–74.8
difference 2 (c-e)	11.4±24.6	−45.2–60.1
difference 3 (d-f)	20.0±33.2	−64.7–101.8

The values are presented as the means±SD (µm).

The values are slightly different from those in Table 2 because these are the raw data.

**Table 4:** The results of symmetrical comparisons between two sectors in the glaucoma group.

Comparison between two sectors	P value
superior vs inferior ave	0.001
superior inside vs inferior inside ave	<0.001
superior outside vs inferior outside ave	<0.001
superior inside vs outside ave	<0.001
inferior inside vs outside ave	0.004

Ave, average.

All p values were obtained using the paired t-test.

### The results of a stepwise regression analysis of independent predictors of the choroidal thickness, and the differences in the choroidal thickness values in all symmetrical sectors among the glaucoma subjects

Table 5 shows the results of the stepwise analyses to determine the predictors of the choroidal thickness using the previously reported factors; age, axial length, CCT and MD. According to these analyses, the age was found to be the most predictive and significant factor for the choroidal thickness in all seven sectors (P<0.001). The axial length was the second most predictive factor and was found to be a statistically significant predictor in almost all sectors. The CCT was identified as the only predictive factor in the symmetrical comparisons; however, all adjusted R2 values in the symmetrical comparison were low (0.037–0.063) and were not statistically significant (all p>0.05). The MD value was not identified as a significant factor in any sector or in the symmetrical comparisons.

**Table 5:** The results of the stepwise regression analyses of the choroidal thickness values using the age, axial length, CCT and MD as variables in the glaucoma group.

	Independent variables												
	Age			Axial length			CCT			MD value			
Dependent variables	Coefficient	F value	P value	Coefficient	F value	P value	Coefficient	F value	P value	Coefficient	F value	P value	Adjusted R^2^
The central sector	**−4.091**	**17.718**	**<0.001**	**−23.458**	**5.646**	**0.022**	-			-			0.287
a: superior field average	**−3.309**	**18.515**	**<0.001**	**−16.963**	**4.715**	**0.036**	-			-			0.297
b: inferior field average	−**3.237**	**19.713**	**<0.001**	**−15.246**	**4.239**	**0.046**	-			-			0.313
c: superior inside average	**−3.568**	**16.545**	**<0.001**	**−22.536**	**6.369**	**0.015**	-			-			0.274
d: superior outside average	**−3.191**	**20.158**	**<0.001**	−13.531	3.510	0.069	0.325	1.753	0.193	-			0.318
e: inferior inside average	**−3.418**	**15.629**	**<0.001**	**−21.267**	**5.863**	**0.020**	-			-			0.261
f: inferior outside average	**−3.091**	**22.245**	**<0.001**	−10.428	2.454	0.125	-			-			0.355
Difference 1 (a-b)	-			-			0.275	3.631	0.064	-			0.063
Difference 2 (c-e)	-			-			0.212	2.533	0.119	-			0.037
Difference 3 (d-f)	-			-			0.329	3.395	0.073	-			0.057

-: excluded variables.

Statistically significant independent factors for the dependent variables are shown in bold.

## Discussion

In the present study, we first reported that the whole macular choroidal thickness values in glaucoma subjects do not differ significantly from those of normal subjects. Previous reports have only shown pinpoint measurements of the choroidal thickness obtained using the enhanced-depth imaging method; however, by using SS-OCT, we were able to analyze the whole macular choroidal thickness and compare different sectors. The subjects evaluated in the present study exhibited only focal or diffuse inferior optic rim thinning with corresponding superior visual field damage. We first assumed that in such patients, the inferior macular choroidal thickness might be thinner due to the glaucoma-related damage compared with the superior macular choroidal thickness or the thickness in normal subjects.

However, no significant differences in the macular choroidal thickness were found between glaucoma patients and normal subjects in any of the sectors (All p>0.4). Furthermore, our results obtained by a stepwise regression analysis showed that the differences in the choroidal thickness values observed between the superior and inferior sectors had no association with glaucoma damage (MD value), suggesting that the secondary changes due to the progression of glaucoma may not extend to the choroid. This observation was also confirmed by the results of a comparison among sectors in the glaucoma patients (Table 4). The glaucoma patients had thicker values in the superior field than in the inferior field and in the temporal field (outside sector) compared to the nasal field (inside sector) in our study, similar to the findings observed in normal subjects [4], [5], [18]. The macular choroidal thickness value was affected by age and the axial length, but not the CCT or MD values, according to the stepwise regression analysis performed in this study. Therefore, this result suggests that the whole macular choroidal thickness values of our glaucoma patients were similar to those obtained in normal subjects, as demonstrated in previous reports [5], [7], [8]. Moreover, the values were not affected by either glaucoma itself or by glaucoma visual field progression.

Both glaucoma patients and normal subjects had a decreased choroidal thickness in inferior sections (Table 2), which is supported by the data form normal adults and children reported by Nagasawa et al. [12]. In addition, the initial changes in glaucoma frequently occur in the inferior optic disc rim, with superior visual field defects being found in glaucoma patients. It has been speculated that inferior choroidal thinning plus some other factors (not choroidal thinning alone) might be associated with the initial glaucomatous changes. However, no mechanism responsible for these changes has been proven. Ikuno et al., advocated two mechanisms underlying the inferior choroidal thinning in normal subjects; one is the choroidal watershed, which isolates the choroidal circulation, and the other is the fetal choroidal fissure, which closes inferiorly at 16 weeks [19]. It also remains unclear as to why the initial changes due to glaucoma occur in the inferior rim; although gravity may be the simplest explanation.

In a comparison of the group characteristics, our glaucoma patients included more females than the normal control group (P<0.01). This was likely due to patient selection, because typical notches or rim thinning are usually observed in patients with focal ischemic type discs, as classified by Nicolela and Drance [20]. They classified the glaucoma disc morphology into four subtypes: focal ischemic, myopic glaucomatous, senile sclerotic and generalized enlargement. The focal ischemic type is most commonly observed in female patients with primary open angle glaucoma [20], and similar findings have been noted in patients with normal tension glaucoma (NTG) [21].

However, Roberts et al. reported that there was a relationship between these glaucoma optic disc types and the peripapillary choroidal thickness values, in that patients with focal ischemic and generalized enlargement exhibited no statistically significant differences compared to healthy subjects [22]. No significant differences were found in the central sector choroidal thickness between the normal females and males (208.9 µm vs 218.6 µm, P = 0.678 by Student's *t*-test). Furthermore, no significant differences were found in the central sector choroidal thickness between female and male glaucoma patients (216.7 µm vs 242.6 µm, P = 0.347 according to Student's t-test). These data support our results, and previous reports have shown that sex is not a useful factor for predicting the choroidal thickness [6], [8].

Other assessments to clarify the relationship between glaucoma and the choroidal thickness have been carried out by investigating the choroidal thickness values in peripapillary areas [6], [22]–[24]. Because the blood supply of the prelaminar region of the optic nerve head is supplied by peripapillary choroidal vessels, and because the laminar cribrosa is supplied by the arterial circle of Zinn or the branches of the posterior ciliary arteries that supply the choroid [25], investigating the relationship between the peripapillary choroidal thickness and glaucoma is interesting. However, Maul et al. reported that the peripapillary choroidal thickness values were not significantly different between glaucoma and suspected glaucoma patients, and were not associated with glaucoma-related damage or the RNFL thickness [6]. Ehrlich et al. [23]. also reported that the peripapillary choroidal thickness values were not associated with the glaucoma damage, the RNFL thickness or the zone of β clock hours parapapillary choroidal atrophy, which are associated with glaucoma damage [26], in either glaucoma subjects or suspected cases. In addition, Suh et al. used spectral-domain OCT and reported that the peripapillary choroidal thickness values correlated with the subject age and axial length, but not with the CCT, MD, IOP or the presence of systemic disease [24].

These previous reports showed no apparent evidence of a relationship between glaucoma and the peripapillary choroidal thickness, which supports our results showing that neither glaucoma-related visual damage nor glaucoma itself have any apparent associations with the whole macular choroidal thickness.

Meanwhile, Usui et al. hypothesized that the 50% thinning in the choroidal thickness values observed in patients with highly myopic NTG results in reduced choroidal circulation, which may be associated with narrowing of the posterior ciliary arteries due to axial length elongation [11]. The differences in the anatomical structure caused by an abnormal refractive error may account for the differences in conclusions between the present and that study [11].

One limitation of the present study is that the adjusted R^2^ values obtained in the stepwise analysis were <0.4. The age and axial length are representative predictive factors for the CT value. However, Aksoy et al. reported that the choroidal thickness is affected by more factors than was previously estimated; for example, the diurnal choroidal thickness changes occur at 30–60 µm, and the use of intravenous acetazolamide increases the CT values [27]. Therefore, we measured the choroidal thickness values during a limited time period (from 1:00 PM to 5:00 PM), and patients receiving an oral topical carbonic inhibitor (acetazolamide) were excluded. However, some patients use topical carbonic inhibitors, which can be absorbed through the mucous membranes in the nose, which then enter the intravenous circulation. In addition, topical prostaglandin analogues and beta blockers have the potential to affect the choroidal thickness. Furthermore, the systolic blood pressure values [16] and hypercholesterolemia [28] may also affect the choroidal thickness; however, these parameters were not examined in this study.

A second limitation is that this study included a relatively small number of subjects. However, it fulfilled a strict power analysis to detect a 20% difference in the choroidal thickness (40 µm) between glaucoma subjects and normal subjects compared with previous reports of 63 µm [5], [8]. A third limitation is that whether the choroidal thickness truly represents the choroidal blood flow remains to be elucidated. In healthy younger subjects, the choroidal thickness was not associated with either the total choroidal blood flow or the subfoveal choroidal blood flow [29]. However, systemic administration of sildenafil citrate (Viagra, Pfizer, New York, NY) has been reported to increase the choroidal blood flow and the choroidal thickness in healthy subjects [30]. Further studies are therefore necessary to clarify the relationship between the choroidal thickness and the choroidal blood flow.

The fourth limitation is that we used manual segmentation techniques, according to previous reports. However, Mansouri et al. recently reported that the findings of automated segmentation obtained using SS-OCT are not affected by operator effects, and exhibit a high level of repeatability, and that the main artifact is blinking, not segmentation errors [31]. The fifth limitation associated with this study is that SS-OCT could cover only 20 degrees in the macula area which thus correlated with the first and second test-points in the 30-2 test program. The 10-2 test program was too small for the region and could not reflect the glaucoma visual field damage, therefore the 24-2 test program might closely correlate with the macula area obtained by SS-OCT. In summary, this study demonstrated that neither glaucoma-related visual damage nor glaucoma itself has any apparent association with the whole macular choroidal thickness.

## Supporting Information

Table S1 **The 3D-OCT values in the glaucoma group.** All p values were obtained using the paired t-test. The values are presented as the mean±SD (µm).
(DOCX)

Data S1 (XLSX)
