# Supplementary material for: The Whole Macular Choroidal Thickness in Subjects with Primary Open Angle Glaucoma
Source: PLoS One. 2014 Oct 28;9(10):e110265. doi: 10.1371/journal.pone.0110265 (PMC4211920; doi:10.1371/journal.pone.0110265)
Supplement: Table S1 — The 3D-OCT values in the glaucoma group. All p values were obtained using the paired t-test. The values are presented as the mean±SD (µm). (DOCX) [file pone.0110265.s001.docx]

|  | Superior hemi field | Range | Inferior hemi field | Range | P value |
| --- | --- | --- | --- | --- | --- |
| RNFL | 35.2±4.1 | 30.0-45.0 | 18.3±7.4 | 3.4-31.1 | <0.0001 |
| GCL+; ganglion cell layer (GCL)/inner plexiform layer (IPL) thickness | 69.3±4.1 | 60.9-77.7 | 57.4±4.8 | 48.2-69.9 | <0.0001 |
| GCL++; macular RNFL+GCL+IPL. | 104.7±6.6 | 92.1-122.0 | 75.7±8.8 | 55.7-94.9 | <0.0001 |
